# Antioxidant, Anti-Inflammatory, and Anti-Apoptotic Effects of *Azolla pinnata* Ethanolic Extract against Lead-Induced Hepatotoxicity in Rats

**DOI:** 10.3390/antiox9101014

**Published:** 2020-10-19

**Authors:** Ahmed Shaaban Abd Elrasoul, Ahmed Abdelmoniem Mousa, Sahar Hassan Orabi, Mostafa Abd El-Gaber Mohamed, Shaban M. Gad-Allah, Rafa Almeer, Mohamed M. Abdel-Daim, Shaden A. M. Khalifa, Hesham R. El-Seedi, Mabrouk Attia Abd Eldaim

**Affiliations:** 1 Department of Biochemistry and Chemistry of Nutrition, Faculty of Veterinary Medicine, University of Sadat City, Sadat City, Menoufia 32897, Egypt; ahmed.phd@yahoo.com (A.S.A.E.); ahmed.mousa@vet.usc.edu.eg (A.A.M.); saher977@yahoo.com (S.H.O.); 2 Department of Pathology, Faculty of Veterinary Medicine, Menoufia University, Menoufia, 32512, Egypt; mostafaabdelgaber@vet.menofia.edu.eg; 3 Department of Surgery, Faculty of Veterinary Medicine, University of Sadat City, Sadat City 32958, Egypt; Shaban.gadallah@vet.usc.edu.eg; 4 Department of Zoology, College of Science, King Saud University, P.O. Box 2455, Riyadh 11451, Saudi Arabia; ralmeer@ksu.edu.sa (R.A.); abdeldaim.m@vet.suez.edu.eg (M.M.A.-D.); 5 Pharmacology Department, Faculty of Veterinary Medicine, Suez Canal University, Ismailia 41522, Egypt; 6 Department of Molecular Biosciences, The Wenner-Gren Institute, Stockholm University, SE-106 91 Stockholm, Sweden; shaden.khalifa@su.se; 7 Department of Chemistry, Faculty of Science, Menoufia University, Shebin El-Kom 32512, Egypt; 8 International Research Center for Food Nutrition and Safety, Jiangsu University, Zhenjiang 212013, China; 9 Department of Biochemistry and Chemistry of Nutrition, Faculty of Veterinary Medicine, Menoufia University, Shebin El-Kom, Menoufia 32512, Egypt

**Keywords:** lead acetate, hepatotoxicity, *Azolla pinnata*, caspase 3, NMR, LC–MS-MS, TNF-α, IL-1β, IL-10

## Abstract

The current study investigated the protective potential of *Azolla pinnate* ethanolic extract (APE) against lead-induced hepatotoxicity in rats. Sixty male Wistar albino rats were randomly allocated into six groups (n = 10). The control group was orally administrated with saline. The second group received lead acetate (100 mg/kg body weight (BW) orally for 60 days). The third group was fed with APE (10 mg/kg BW orally for 60 days). The fourth group was administrated with lead acetate like the second group and APE like the third group, concomitantly, for 60 days. The fifth group was administrated with APE like the third group for 30 days, then orally administrated with the lead acetate like the second group for another 30 days. The sixth group was administrated with lead acetate like the second group for 30 days, then with APE like the third group for a further 30 days. Phytochemical analysis of APE indicated the presence of peonidin 3-*O*-glucoside cation, vitexin, rutin, thiamine, choline, tamarixetin, hyperoside, astragalin, and quercetin. The latter has been elucidated using one- and two-dimensional nuclear magnetic resonance (1D and 2D NMR) and liquid chromatography–mass spectrometry (LC–MS-MS). Lead acetate increased the serum levels of alanine and aspartate aminotransferases and that of urea, creatinine, tumor necrosis factor alpha, and interleukin 1*β*, hepatic tissue malondialdehyde contents, and caspase 3 protein expression, as well as altering the hepatic tissue architecture. However, it decreased the serum levels of interleukin 10 and glutathione (GSH) contents, and the activities of catalase and superoxide dismutase in hepatic tissue. In contrast, the administration of APE ameliorated the lead-induced alterations in liver function and structure, exemplifying the benefits of *Azolla*’s phytochemical contents. Collectively, *A. pinnate* extract is a protective and curative agent against lead-induced hepatotoxicity via its antioxidant, anti-inflammatory, and anti-apoptotic impacts.

## 1. Introduction

The main cause of hepatotoxicity in all living organisms is exposure to heavy metals, toxins, drugs, or harmful compounds, including carbon tetrachloride, sodium oxalate, and ethylene glycol [1]. Lead acetate is a white crystalline chemical compound found under the earth’s crust and has a sweet taste [2]. The contamination of air, water, soil, food by paints, disposable materials of factories like batteries and leaded gasoline is the main reason for lead poisoning [3,4]. Water is an important source for lead poisoning, particularly due to the leaking of lead from water pipes [5]. Lead acetate induces experimental hepatic injury in rats via the induction of oxidative stress following an imbalance between free radical generation and the antioxidant defense system [6]. This oxidative stress leads to the generation of reactive oxygen species (ROS), including the hydroperoxides, singlet oxygen, and hydrogen peroxide, resulting in serious damage to different biomolecules, i.e., DNA, enzymes, proteins, and membrane lipids. It also impairs homeostasis simultaneously [6,7]. Lead generates free radicals that damage the vital organs, including the liver [8], via reducing the activities of antioxidant enzymes and increasing lipid peroxidation [9]. Furthermore, lead toxicity has an inferior impact on various organs, including the nervous system, bones, teeth, kidneys, cardiovascular, immune, and reproductive systems [10], usually leading to hearing loss and tooth decay [11,12,13]. Lead toxicity also results in neuropsychiatric disorders ranging from headache, difficulty concentrating, and delayed motor nerve conduction, as well as to delayed reaction times and irritability [14]. Furthermore, exposure to higher levels of lead causes encephalopathy, characterized by swelling of the brain tissue associated with delirium, coma, and seizures [15]. Chronic lead toxicity results in short-term memory loss, nausea, depression, loss of coordination, numbness and tingling in the extremities, and abdominal pain [16], in addition to anemia [14]. Lead toxicity harms both adults and children [17].

Medicinal plants possess an important role in the human healthcare system. Herbal medicine has received tremendous attention in the primary health sector, mainly due to its effectiveness and popularity [18]. In traditional medicine, herbal prescriptions were claimed to be effective in treating liver disorders; hence, the development and validation of a new herbal drug is of prime concern [19].

*A. pinnata* is the only genus in the family Salviniaceae that has a worldwide distribution; it can tolerate both temperate and tropical climates. *A. pinnata* is a species of fern that has several common names, including mosquito fern, feathered mosquito fern, and water velvet. It is native to Africa and Asia [20]. It is an aquatic fern consisting of a short, branched, floating stem, with bearing roots hanging down in the water. The leaves are alternately arranged; each consists of a thick aerial dorsal lobe containing green chlorophyll and a thin, floating ventral colorless lobe of a slightly larger size [21]. *A. pinnata* contains many bioactive compounds such as essential amino acids, vitamins, Beta-carotene, minerals, saponin, and flavonoids [22]. It is also considered a good source of high-quality protein [23]. To investigate further the protective effect of *A. pinnata* ethanolic extract against lead acetate-induced hepatotoxicity in rats, we elucidated the underlying molecular mechanism as part of our ongoing project of phytochemical analysis of Egyptian plants, with particular emphasis on the antioxidant, anti-inflammatory and anti-apoptotic activities [24].

## 2. Materials and Methods

### 2.1. Animals

A total of 60 male Wistar albino rats, weighing 90–110 g each, were purchased from Vac Sera lab., Helwan City, Cairo, Egypt. The rats were kept in polypropylene cages under standard laboratory conditions of temperature 20–25 °C and a 12 h light/12 h dark cycle. Rats were provided with clean water and had access to food ad libitum. A balanced diet of commercial pellets was fed to the rats (Atmida Company for international commerce and development, Egypt). The rats were kept for 10 days before the beginning of the experiments for acclimatization. Animal rearing and handling, and the experimental design and procedures, were approved by the Research Ethics Committee of the Faculty of Veterinary Medicine, University of Sadat City, Egypt (VUSC-006-2-20).

### 2.2. Chemicals

All chemicals used in this study were of analytic grades.

### 2.3. Preparation of Plant Extract

For the preparation of *A. pinnata* ethanolic extract (APE), fresh green leaves of *A. pinnata* were obtained from the botanical gardens of the National Research Centre, Giza, Egypt. The extract was prepared according to the methods of Kunjiappan et al. (2014) [25]. Briefly, fresh leaves of *A. pinnata* were collected and dried under shade at room temperature (22 °C and 65% relative humidity) for 7 days. The dried leaves were grounded into powder and soaked in ethanol 70% (500 g/L) for 48 h with gentle shaking in an incubator shaker at 37 °C. The content was filtered through a Whatman No. 1 paper filter, and the filtrate was evaporated until dryness. The yield was 10%, and the content was kept in an airtight bottle in a refrigerator at 4 °C until usage.

### 2.4. NMR Analysis

^1^H-NMR spectra and heteronuclear single quantum coherence spectroscopy (HSQC) spectra were recorded at 298 K on a Bruker 600 MHz spectrometer (TCI CRPHe TR-^1^H and ^19^F/^13^C/^15^N 5 mm-EZ CryoProbe). Chemical shifts were referenced to the solvent peaks for (CD_3_)_2_SO at δ_H_ 2.50 and δ_C_ 39.52.

### 2.5. UPLC–MS-QToF Analysis and Molecular Networking for a Screening of Secondary Metabolites

High-resolution mass spectrometric measurements were acquired using a ultra-performance liquid chromatography coupled with quadrupole time-of-flight mass spectrometry (UPLC-QToF) nanospray MS (Waters nanoAcquity, QToF Micro). The UPLC column used was a Waters ACQUITY UPLC M-Class Peptide BEH C18 column (1.7 μm, 130 Å, 75 μm × 150 mm), using solvents A (0.1%), FA (Formic acid) in water and B (0.1% FA in AcN). A total run of 75 mins with a flow rate of 0.3 µL/min is used as follows. A gradient from 1–90% of AcN in 50 min, washing the column with 90% AcN for 4 min, and finally, equilibration of a column to 1% AcN again in 20 min. MS and MSMS data were acquired in positive electro spray ionization mode (+ESI), nanoelectrospray (NSI) is at 90°. The mass range was from 50–2000 Da/ms and 50–1300/msms, the scan time was 1 s, and the inter-scan delays were 0.1 s. The collision gas, argon, spray energy 4300 V collision energy is 30 volts, the sample cone is 30 V, the extraction cone is 2.5 V, the source temperature is 80 °C and the desolvation temperature is 100 °C. Furthermore, molecular networking provides a valuable tool in metabolite identification by grouping metabolites with the same fragmentation in clusters and linking them to each other; so, if some metabolites are identified, the whole cluster can be identified from mass loss. Moreover, it can give information on the similarities and variances among the species that have been subjected to MS fragmentation.

### 2.6. Experimental Design

A total of 60 male Wistar albino rats were equally divided into 6 groups, (n = 10).

#### 2.6.1. Control Group

Rats were orally given normal physiological saline (0.9% sodium chloride).

#### 2.6.2. Lead Acetate Group

Lead acetate was orally administered to rats at a dose of 100 mg/kg body weight (BW) for 60 days [26].

#### 2.6.3. *A. pinnata* Ethanolic Extract (APE) Group

Rats were orally administered with 10 mg/kg BW of APE, extracted daily for 60 days [27].

#### 2.6.4. Lead Acetate and *A. pinnata* Ethanolic Extract Group

Rats were orally administrated with lead acetate as the second group, and APE as the third group, simultaneously for 60 days.

#### 2.6.5. *A. pinnata* Ethanolic Extract then Lead Acetate Group

Rats were orally administrated with APE as the third group for 30 days, then administrated with the lead acetate as the second group for another 30 days.

#### 2.6.6. Lead Acetate then *A. pinnata* Ethanolic Extract Group

Rats were orally administrated with lead acetate as the second group for 30 days, then administered with APE as the third group for further 30 days.

### 2.7. Recording the Initial and Final Rats’ Weights

Rats’ initial and final weights were recorded at the beginning and the end of the experiment, respectively.

### 2.8. Blood and Tissue Sampling

At the end of the experiment, rats were anaesthetized, then blood samples were collected from the medial canthus of the eye with a heparinized capillary tube. Sera samples were separated and stored at −20 °C to be used for measuring the biochemical parameters. The liver was removed and divided into two parts. The first part was kept at −80 °C for further investigation of lipid peroxidation and antioxidant activity biomarkers. The second part was kept in 10% neutral formalin to be used for histopathological and immunohistochemical investigations.

### 2.9. Biochemical Assays

Liver and kidney function biomarkers were determined by using specific commercial diagnostic kits. The serum concentration of urea was measured according to the methods of Fawcett et al. (1960) [28] and serum creatinine concentrations were measured according to the methods of Bartels et al. (1972) and Larsen et al. (1972) [29,30], according to the manufacturer’s instructions. Serum alanine aminotransferase (ALT) and aspartate aminotransferase (AST) activities were analyzed according to the methods of Reitman et al. (1957) [31].

Malondialdehyde (MDA) was determined in liver homogenate according to the procedure described by Satoh, 1978 [32], using Biodiagnostic Kits for MDA. Reduced glutathione (GSH) concentration was determined in liver homogenate, according to the procedure described by Beutler et al. (1963) [33], using Biodiagnostic Kits. Superoxide dismutase (SOD) activity was determined in liver homogenate, according to the procedure described by Nishikimi et al. (1972) [34], using Biodiagnostic Kits for SOD. Catalase activity was determined in liver homogenate according to the procedure described by Fossati et al. (1980) and Aebi (1984) [35,36], using Biodiagnostic Kits.

Serum levels of interleukin 1 beta (IL-1 β), tumor necrosis factor alpha (TNF-α), and interleukin 10 (IL-10) were determined by using ELISA kits according to methods described by Vidal et al. (2000), Brynskov et al. (2002), and Odewumi et al. (2015), respectively [37,38,39].

### 2.10. Histopathological Examination

Following necropsy, liver tissue samples were collected and fixed in 10% neutral buffered formalin for 3 days. Fixed samples were routinely processed, embedded in paraffin wax, cut into 4-µm sections, and stained with hematoxylin and eosin (H&E) [40]. Five fields per section were examined for the evaluation of hepatic damage. The severity of pathological findings was assessed by using a modified semiquantitative scoring system (- means no changes were present, + means mild changes, ++ means moderate, and +++ means severe changes) [41,42].

### 2.11. Immunohistochemical Analysis

The immunohistochemical staining was done according to the methods of Orabi et al. (2020) [43]. Rabbit polyclonal caspase 3 primary antibodies (abcam, abc2302; 1:100 dilution) and anti-rabbit IgG secondary antibodies (EnVision + System HRP; Dako) were used. Diaminobenzidine commercial kits (Liquid DAB+Substrate Chromogen System; Dako) were used to visualize the stained caspase 3; finally, slides were counterstained with Mayer’s hematoxylin. The negative control procedure was carried out using the aforementioned procedures, while the primary antibody was replaced by normal rat serum. The labeling index of caspase 3 was expressed as the percentage of positive cells per 1000 counted cells in about 10 high-power fields.

### 2.12. Statistical Analysis

An analysis of the results was performed by using SPSS program software, version 16 (IBM^®^, USA). Data were subjected to an analysis of variance (ANOVA) and Duncan’s post-hoc tests to determine significant differences among the data. The differences between means were analyzed at the 5% probability level (*p* ≤ 0.05), which was statistically significant.

## 3. Results

### 3.1. Nuclear Magnetic Resonanse (NMR) Analysis

**Figure 1:**
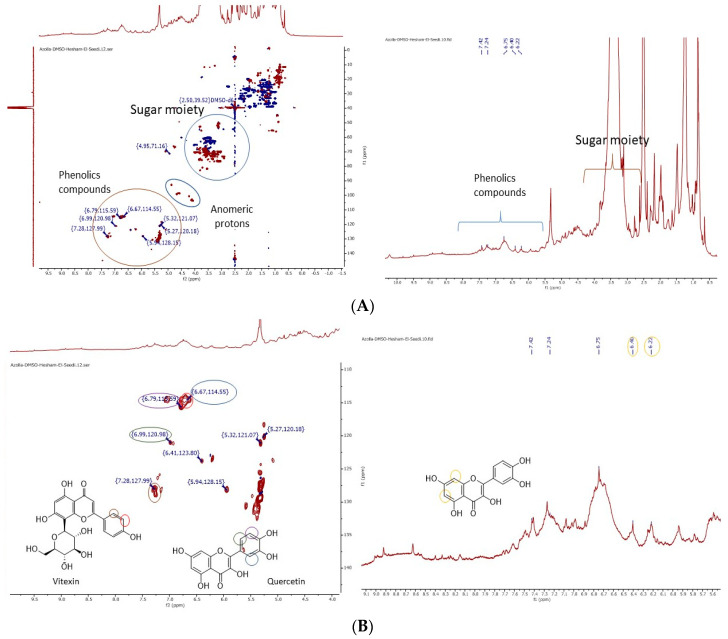
(**A**) ^1^H-NMR and heteronuclear single quantum coherence spectroscopy (HSQC) spectrum of A. pinnata ethanolic extract. (**B**) ^1^H-NMR and HSQC spectrum of *A. pinnata* ethanolic extract that prescribes the phenolic areas of quercetin and vitexin.

The ^1^H-NMR spectra of *A. pinnata* ethanolic extract showed a dominance of signals in the aliphatic region (e.g., methyl proton peak at δ 0.9, methylene protons [CH_2_]_n_ peaks in the region of δ 1.2–3.5 ppm). Particular glycosides (e.g., anomeric hydrogen at δ 4.8 ppm) and some of the aromatic moieties at δ (6.00–8.2 ppm) are presented in Figure 1A. Heteronuclear single quantum coherence spectroscopy (HSQC) with a decoupled sensitive spectrum prescribed the presence of glycosides, CH groups as red spots at a range of δ 60–80 ppm, and SP2 methylene groups as blue spots with a range of δ 54–66 ppm. Additionally, anomeric proton CH groups can be seen as red spots around chemical shifts at δ 100 ppm. Kaempferol and vitexin are flavanones characterized by the presence of the 1,4 di-substitution benzene ring H-2′-3′ at δ 7.24 and 6.67 and ^13^C δ 127 and 114 ppm, respectively. Quercetin is characterized by the ABX, A-B is in ortho position and A-X is in meta position, benzene ring H-2′-3′ and 6′ at δ 6.99, 6.79, and 6.67, with ^13^C δ 120, 117, and 114 ppm, respectively, as seen in Figure 1B.

### 3.2. UPLC–MS-QToF and Molecular Networking Analysis

**Figure 2:**
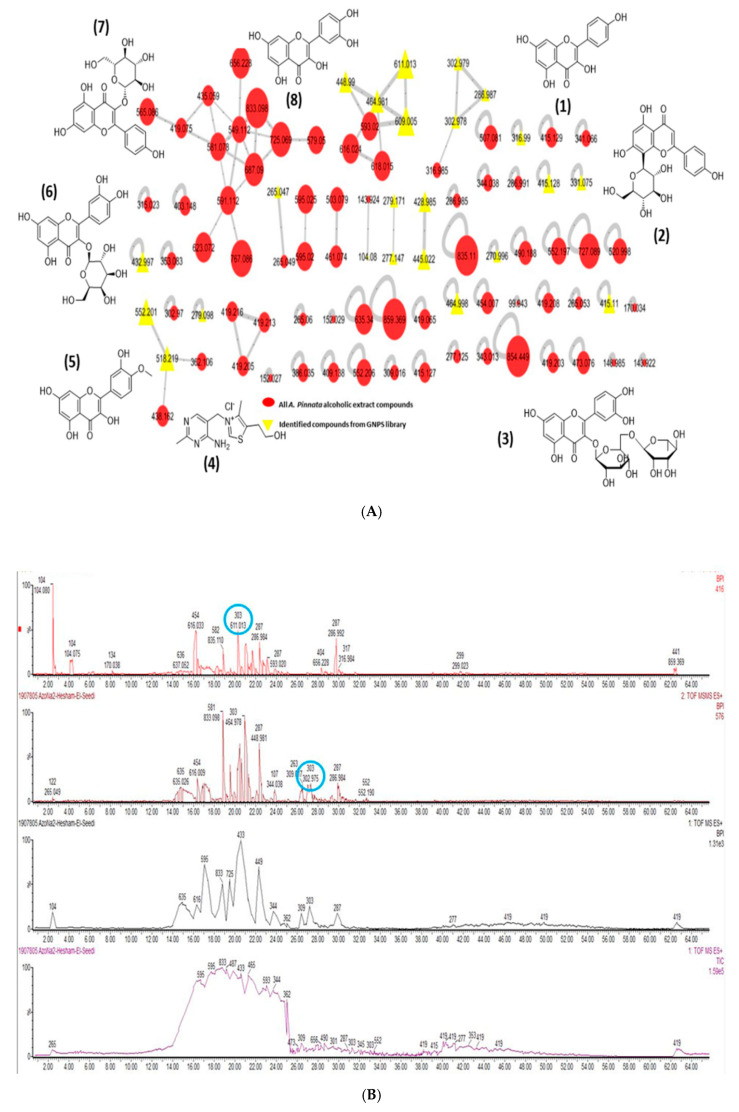
(**A**) Base peak MS chromatogram showing the major components of *A. pinnata*. (**B**) *A. pinnata* metabolite parent masses molecular network. The red circular nodes refer to all metabolite parent masses revealed from raw mass spectra. The yellow triangle nodes are for the identified metabolites from the Global Natural Product Social Molecular Networking (GNPS) library.

The mass profiling molecular networking for the metabolites of the species *A. pinnata* presented 89 nodes for 89 parent masses, as seen in Figure 2.

All the nodes are reflections of metabolites with unique peaks in the raw mass spectra. The bigger the node size, the higher the intensity of the metabolites. The intensity indicates the number of the compounds’ ions in the extract. Five clusters for chemically related metabolites with common classes are detected in the molecular network. Out of 89 metabolites, only 22 were identified based on the molecular networking database. Eight metabolites were found to be reliable and are considered, as shown in Table 1 [44].

*A. pinnata* ethanolic extract was found to be rich in flavonoids. These flavonoids were identified using molecular networking (GNPS) and prescribed as follows: kaempferol with *m*/*z* 286.987 [C_15_H_10_O_6_]^+^ [M + H]^+^ and its glucoside derivative, i.e., *m*/*z* 448.99 [C_21_H_20_O_11_]^+^ by loss of glucoside (−162 amu), annotated as kaempferol-*O*-glucoside (astragalin); a flavonoid with *m*/*z* 302.979 [C_15_H_10_O_7_]^+^ [M + H]^+^, reported as quercetin, and its glucoside moieties with *m*/*z* 464.91 [C_22_H_23_O_11_]^+^ by loss of glucoside (−162 amu), annotated as quercetin-*O*-glucoside (hyperoside), and loss of two glucoside units (−324 amu) *m*/*z* 611.013 [C_27_H_30_O_16_]^+^ [M + H]^+^, annotated as rutin; vitexin *m*/*z* 432.997 [C_21_H_20_O_10_]^+^ [M + H]^+^, which is the flavone C-glycoside. Additionally, vitexin was revealed to exert notable antioxidant activity against 5-(6-)chloromethyl-2′,7′-dichlorodihydrofluorescein diacetate (CM-H2DCFDA), and robust free radical scavenging activity in UVB-irradiated human dermal fibroblasts. We also identified the following: amarixetin *m*/*z* 316.99 [C_16_H_12_O_7_]^+^ [M + H]^+^; peonidin-*O*-glucoside *m*/*z* 464.998 [C_22_H_23_O_11_]^+^ [M + H]^+^; a flavonoid with *m*/*z* 609 [C_27_H_31_O_16_]^+^ [M + H]^+^ with two glucoside moieties (−324 amu), annotated as delphinidin-3-rutinoside.

### 3.3. A. pinnata Extract Normalized Lead Acetate Reduced Final Body Weight of Rtas

Table 2 shows the initial and final weights of the rats of different experimental groups. The intoxication of rats with lead acetate significantly (*p* < 0.05) decreased the final weights compared with those of the control group. However, the administration of lead acetate-intoxicated rats with APE (fourth, fifth, and sixth groups) normalized body weights. On the other hand, APE itself had no significant effect on rats’ final body weights (Table 2).

### 3.4. A. pinnata Extract Modulated the Toxic Effects of Lead Acetate on Liver and Kidney Functions Biomarkers

The intoxication of the rats with lead acetate significantly elevated (*p* < 0.05) the activities of serum ALT and AST, and serum levels of urea and creatinine compared with the control group. However, the supplementation of rats with APE—before, during, or after their intoxication with lead acetate (fourth, fifth or sixth groups)—significantly reduced the lead acetate-elevated activities of serum ALT and AST, and serum levels of urea and creatinine compared with rats intoxicated with lead acetate only (second group) (*p* < 0.05). Treating rats with APE (third group) had no significant effect on liver and kidney function biomarkers compared with the control rats (first group) (*p* < 0.05) (Table 3).

### 3.5. A. pinnata Extract Reversed the Effects of Lead Acetate on Serum Levels of Inflammatory and Anti-Inflammatory Cytokines

The effects of lead acetate and/or APE on serum levels of IL-1β, IL-10, and TNF-α are presented in Table 3. The administration of rats of the second group with lead acetate significantly elevated (*p* < 0.05) serum levels of IL-1β and TNF-α, while it reduced serum levels of IL-10 compared with the control rats. However, treating lead acetated-intoxicated rats (fourth, fifth and sixth groups) with APE significantly reduced (*p* < 0.05) the elevated serum levels of IL-1β and TNF- α, while it increased serums level of IL-10 compared with the lead acetate-intoxicated group (second group). Supplementation of rats with APE (third group) significantly elevated (*p* < 0.05) serum levels of IL-10, while it had no significant effects on serum levels of IL-1β and TNF-α compared with the control rats (Table 4).

### 3.6. A. pinnata Extract Ameliorated the Deleterious Effects of Lead Acetate on Oxidative/Antioxidant Status in Hepatic Tissues of Rats

The effects of lead acetate and/or APE on the hepatic tissue lipid peroxidation and antioxidant defense system biomarkers of rats are shown in Table 4. Orally administrated lead acetate significantly increased the hepatic tissue level of MDA (*p* < 0.05) compared with the normal control rats. However, treatment of lead acetate-intoxicated rats (fourth, fifth, and sixth groups) with APE significantly reduced MDA contents in the hepatic tissue (*p* < 0.05) compared with lead acetate-intoxicated group (second group). In contrast, lead acetate significantly reduced GSH contents, SOD, and CAT activities in hepatic tissues of the second groups compared with the control group. However, supplementation of lead acetate-intoxicated rats with APE significantly elevated GSH contents, SOD, and CAT activities in hepatic tissues of fourth, fifth and sixth groups compared with the rats intoxicated with lead acetate alone (second group). APE itself had no significant effect on MDA and GSH contents, SOD, and CAT activities in hepatic tissues of rats of the second group compared with normal control rats (Table 5).

### 3.7. A. pinnata Extract Ameliorated the Degenerative Effect of Lead Acetate on Hepatic Tissues of Rats

**Figure 3:**
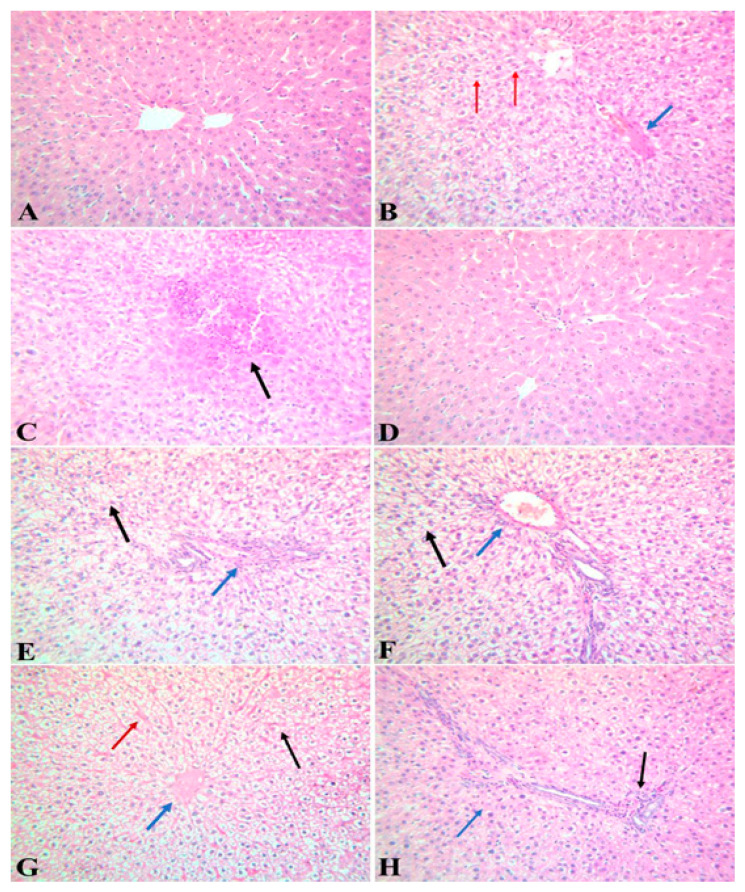
Photomicrographs of liver tissues sections of different groups stained with hematoxylin and eosin (H&E). (**A**) Liver tissue from rats of the control group showed a normal hepatic tissue structure; (**B**,**C**) hepatic tissues of rats from the lead acetate-intoxicated group showed congested blood vessels (blue arrow) and hepatic sinusoids, multiple areas of coagulative necrosis (black arrow) and mononuclear cell infiltrations, as well as the activation and proliferation of Kupffer cells (red arrow); (**D**) liver tissue of rats from the APE group showed normal hepatic tissue; (**E**,**F**) liver tissue of rats intoxicated with lead acetate and treated with APE showed congested blood vessels with perivascular infiltration (blue arrow), while the hepatocytes showed swelling and vacuolar degeneration (black arrow); (**G**) hepatic tissue of rats administrated with APE then intoxicated with lead acetate showed congested blood vessels (blue arrow) and hepatic sinusoids (red arrow) with vacuolation of hepatocytes (black arrow); (**H**) liver tissue of rats intoxicated with lead acetate then treated with APE showed congested blood vessels with perivascular infiltration (black arrow), while the hepatocytes showed single-cell necrosis (blue arrow) (**H** and **E** × 40). The severity of the pathological findings was assessed by using a modified semiquantitative scoring system (- means no changes were present, + means mild changes, ++ means moderate and +++ means severe).

Figure 3 and Table 5 show a histopathological examination of the liver tissues of the different experimental groups. The liver tissues of the control group showed a normal hepatic tissue structure (Figure 3A). The hepatic tissue of the lead acetate-intoxicated group showed a variety of pathological changes represented as severely congested blood vessels and hepatic sinusoids, degenerative changes in the hepatic parenchyma, and multiple areas of coagulative necrosis. Mononuclear cell infiltrations and the proliferation of Kupffer cells were present (Figure 3B,C). A microscopic examination of the hepatic tissue of the APE group showed normal hepatic tissue (Figure 3D). A microscopic examination of the hepatic tissue of rats administrated with lead acetate and the APE group showed moderately congested blood vessels with perivascular infiltration; the hepatocytes showed diffuse swelling and vacuolar degeneration (Figure 3E,F). An examination of hepatic tissue of rats treated with APE, then intoxicated with lead acetate, showed congested blood vessels and hepatic sinusoids with diffuse vacuolation of hepatocytes (Figure 3G). The liver tissue of rats intoxicated with lead acetate, then treated with APE extract, showed congested blood vessels with perivascular infiltration, while the hepatocytes showed single-cell necrosis (Figure 3H).

### 3.8. A. pinnata Extract Reduced Lead Acetate-Induced Caspase 3 Protein Expression in Hepatic Tissues of Rats

**Figure 4:**
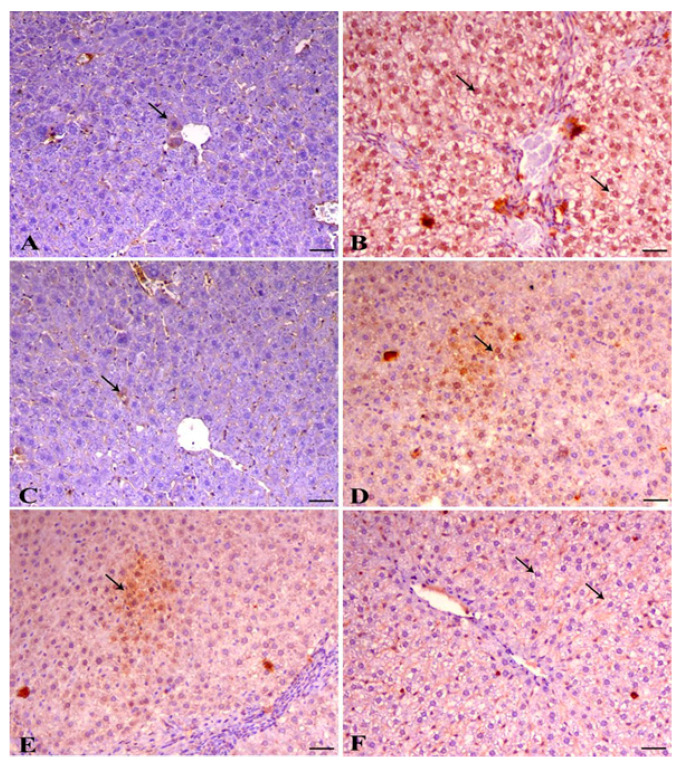
Immunohistochemical analysis of activated caspase 3 in liver tissue of rats. (**A**) Liver of control animal showing mild expression of caspase 3 within the hepatocytes (arrow indicates nuclear expression), cleaved form of caspase 3 IHC, bar = 50 µm, ×200. (**B**) Liver of lead-intoxicated animal showing marked expression of caspase 3 of both cytoplasmic and nuclear expression within the hepatocytes (arrows), cleaved form of caspase 3 IHC, bar = 50 µm, ×200. (**C**) Liver of normal animal treated with *Azolla* showing mild expression of caspase 3 within the hepatocytes (arrow indicates cytoplasmic expression), cleaved form of caspase 3 IHC, bar = 50 µm, ×200. (**D**) Liver of animal treated with both lead and *Azolla* showing decrease the expression of caspase 3 (arrow), cleaved form of caspase 3 IHC, bar = 50 µm, ×200. (**E**) Liver of diseased animal pretreated with *Azolla* showing mild cytoplasmic expression of caspase 3 (arrows), cleaved form of caspase 3 IHC, bar = 50 µm, ×200. (**F**) Liver of diseased animal treated with *Azolla* showing marked decrease in caspase 3 expression within the hepatocytes (arrow), cleaved form of caspase 3 IHC, bar = 50 µm, ×200. The labelling indices of caspase 3 were expressed as the percentage of positive cells per total 1000 counted cells in about 10 high-power fields.

The effects of lead acetate and/or APE on caspase 3 protein expression in the hepatic tissue of rats are shown in Figure 3 and Table 6. The livers of control rats showed a mild expression of caspase 3 within the hepatocytes (arrow indicates nuclear expression) Figure 4A. The oral administration of lead acetate in the second group significantly elevated (*p* < 0.05) the protein expression of caspase 3 in the nucleus and cytoplasm of hepatic tissue compared with the control group (Figure 4B and Table 6). The livers of rats treated with *Azolla* showed a mild expression of caspase 3 within the hepatocytes (arrow indicates cytoplasmic expression) Figure 4C. However, treating the lead acetate-intoxicated rats with APE extract (fourth, fifth and sixth groups) significantly reduced hepatic caspase 3 protein expression compared with rats intoxicated with lead acetate alone (second group) Figure 4D–F and Table 7.

## 4. Discussion

The intoxication of rats with lead acetate decreased the rats’ final body weight. These findings were in line with that of Amjad et al., who indicated that lead induces weight loss in rats [45]. This decrement in the lead acetate-intoxicated rats’ final body weights was caused by the metal toxicity, which induced nausea, vomiting, and anorexia [46] as well as the oxidative stress [47,48,49] that leads dramatically to catabolic states where wasted muscles and cachexia are seen, followed by low body weight [50]. Moreover, lead toxicity has been shown to disrupt hepatic tissue architecture and function [51,52,53]. The results of the current study showed that the intoxication of rats with lead acetate-induced hepatotoxicity, as represented by the elevated activities of serum ALT and AST (Table 3). This finding was in line with those reported by [54], who indicated that lead acetate increases the activities of serum ALT and AST. This finding was explained by the fact that lead acetate induced oxidative stress in hepatic tissues, as it increased the hepatic tissue contents of MDA, while it decreased the hepatic tissue contents of GSH and similarly decreased the activities of SOD and CAT (Table 5). The findings were also in agreement with those discussed by [7], who indicated that lead induces oxidative stress in hepatic tissues as a result of the increase in lipid peroxidation and the antioxidant defense system disturbance in hepatocytes, leading to hepatic injury. Thus, lead acetate and its metabolites induce the redox cycle, with the generation of superoxide radicals and hydrogen peroxide, which subsequently increases lipid peroxidation and decreases antioxidant enzyme activities, resulting in hepatocyte destruction, the activation of innate immunity by producing pro-inflammatory cytokines such as TNF-α and IL-1β in hepatic tissues (Table 4) [55,56]. Lead damages many tissues through the induction of oxidative stress [26,57], leading to lipid peroxidation, which induces inflammatory processes [58]. Moreover, occupational exposure of humans to lead increases serum levels of some pro-inflammatory cytokines [59] such as IL-1, IL-6, and TNF- α [60]. TNF-α is produced at the site of inflammation by activated macrophages and lymphocytes and participates with IL-1β and IL-6 to induce systemic inflammatory reactions [61]. Furthermore, the intoxication of rats with lead acetate in the current study decreased the serum level of the anti-inflammatory cytokine IL-10 (Table 4). This finding was consistent with that of [62,63], who indicated that exposure to lead acetate decreases IL-10 in the rats’ cerebral cortex, confirming the role of leadership in the development of an inflammatory response in rat brain tissue [62]. Such a decrease in IL-10 due to lead acetate exposure may be implicated in the increased serum levels of IL-1β and the promotion of inflammatory signals, as it was shown that IL-10 can block IL-1β gene expression [64]. These inflammatory cytokines may injure the hepatic tissues through activation of the caspase cascade signaling pathway and, as our results revealed, lead acetate intoxication increased caspase 3 protein expression in hepatic tissue. TNF-α has been indicated to induce the apoptosis of hepatocytes through the activation of caspase, while caspase inhibitors block TNF-α production and its signaling pathways [65]. Caspase 3 can be activated in the apoptotic cells either by extrinsic stimuli, including lead acetate, or intrinsic factors inducing mitochondrial stress [66], and it plays important roles in cell apoptosis [67]. In addition, it causes the activation of the innate immunity system by producing pro-inflammatory markers such as TNF-α and IL-1. Furthermore, our study was in accordance with that of [7] who reported that the lead acetate intoxication of rats induces hepatic tissue necrosis and increases serum transaminase activity and hepatic lipid peroxidation. Collectively, lead acetate induced oxidative stress and increased pro-inflammatory mediators, leading to histopathological changes in hepatic tissues (Figure 3), which matched with the results of Arun et al. (2014) [27]. Finally, hepatic tissue injury damages hepatocytes and cause the discharge of liver enzymes, which consequently raises their activities [25].

Regarding the ameliorative effects of the *Azolla pinnata* ethanolic extract against lead acetate-induced hepatotoxicity, our results showed that oral administration of *A. pinnata* decreased the activities of serum ALT and AST and normalized serum levels of urea and creatinine (Table 2). These findings were in line with those of Debashis et al. (2016) [21], who reported that *A. pinnata* reduces serum activities of ALT and AST. The ameliorative effects of *A. pinnata* against lead acetate-induced hepatotoxicity may be attributed to the antioxidant and anti-inflammatory activities of its constituents as 10 metabolites were found to be reliable (Figure 3 and Figure 4, Table 1) [44]. *A. pinnata* ethanolic extract was found to be rich in flavonoids, which are well-known for their antioxidant and anti-inflammatory activities [68]. These flavonoids include kaempferol and its glucoside derivative, namely kaempferol-*O*-glucoside (astragalin). Kaempferol sugar derivatives were studied for their antioxidant and anti-inflammatory scavenging activity for DPPH radicals and protection against oxidative stress-induced cell death [69]. Quercetin and its glucoside moieties are produced by the loss of glucoside annotated as quercetin-*O*-glucoside (hyperoside) or by the loss of two glucoside units that were annotated as rutin [70]. Quercetin and its glucoside moieties were reported to inhibit glioma cell proliferation and migration and modulate inflammatory and growth factors [71]. Vitexin, which is a flavone C-glycoside known for possessing anti-inflammatory activity and inhibiting IL-1β, IL-33 [72], IL-6 and TNF-α [73], was revealed to exert notable antioxidant activity against 5-(6-)chloromethyl-2′,7′-dichlorodihydrofluorescein diacetate (CM-H2DCFDA), and robust free radical scavenging activity in UVB-irradiated human dermal fibroblasts [74]. Tamarixetin exhibited superior anti-inflammatory activity by reducing the secretion of several inflammatory cytokines [73]. These compounds may reduce lipid peroxidation and inflammatory cytokines while increasing antioxidant and anti-inflammatory activities (Table 3 and Table 4), thus consequently modulating lead acetate-induced apoptosis and injury of hepatic tissues (Figure 3 and Figure 4, Table 5 and Table 6). These beneficial effects of APE against lead acetate-induced hepatotoxicity confirmed the findings of Mandal et al. (1993) [66], who indicated that *A. pinnata* suppresses lipid peroxidation and scavenges free radicals, preventing pathological changes in the hepatic tissue architecture [25]. Quercetin normalizes thioacetamide and thus increases the liver biomarkers ALT and AST through the inhibition of p-extracellular signal-regulated kinase (ERK)1/2 and an increase in Bax/Bcl-2 ratio, causing the prevention of cell apoptosis [67]. Moreover, quercetin suppresses prenatal stress–increased serum IL-1β levels via increasing serum IL-10 levels in rats [75]. Thus, the hepatoprotective effect of *A. pinnata* might be attributed to its free radical scavenging activity [76], which suppresses, with the help of Tamarixetin (quercetin 4-methylether), the activity of caspase 3 [77]. The reduction in lead acetate toxicity-induced oxidative stress in our study was not achieved only by quercetin, but also by thiamine [78] as it reduces ethanol-induced oxidative stress in rats by scavenging free radicals or by the regeneration of vitamin E, reducing glutathione levels. Rutin suppresses the activity of pro-inflammatory cytokines through diminishing TNF-α and IL-1β levels [79] and inhibits the hypoxia and excessive glutamate stress-induced caspase 3 expression in retinal ganglion cell [80]. Hence, rutin has hepatoprotective effects against paracetamol, and carbon tetrachloride-induced hepatic toxicity [81]. Hyperoside present in *A. pinnata* has an antioxidant activity by increasing the expression of heme oxygenase-1 (HO-1) and inducing extracellular signal-regulated kinase (ERK) phosphorylation and nuclear factor erythroid 2-related factor 2 (Nrf2) antioxidant response and binding activity, preventing hydrogen peroxide-induced cytotoxicity [72]. Vitexin (Apigenin-8-C-β-D-glucopyranoside) is a chemical compound found in many plants [82] and has a variety of pharmacological activities, including antioxidant [83] and anti-inflammatory effects [84,85]. The antioxidant activity of vitexin is related to its capability to donate electrons, which enables it to act as a good free radical scavenger as it reacts with ROS as 4-OH, 7-OH, and 5-OH [86]. Moreover, the astragalin content of *A. pinnata* in our study may have an anti-inflammatory effect through decreasing serum levels of TNF-α and IL-1β, as it has been indicated that astragalin reduced lipopolysaccharide-induced inflammatory cell infiltration, myeloperoxidase activity and TNF-α, IL-1β and IL-6 expression through inhibiting nuclear factor-kappa B (NF-κB) activation and nuclear factor of kappa light polypeptide gene enhancer in B-cells alpha (IκBα) in the mammary glands of a murine model [87]. Taken together, the possible causes of the hepatoprotective effects of *A. pinnata* against lead acetate-induced hepatotoxicity in our study are the antioxidant, anti-inflammatory and anti-apoptotic activities of *A. pinnata* phytochemical contents as they decreased serum levels of TNF-α and IL-1*β* as well as oxidative stress and caspase 3 protein expression in hepatic tissue, while they increased serum levels of IL-10 and antioxidant activity in hepatic tissues. These activities ameliorated lead acetate-induced histopathological changes in hepatic tissues.

## 5. Conclusions

Lead acetate-induced hepatotoxicity in rats is manifested by oxidative stress, increased pro-inflammatory cytokines, and pro-apoptotic protein in hepatic tissue. However, *A. pinnata* ethanolic extract ameliorated lead acetate-induced hepatotoxicity by reducing oxidative stress and pro-apoptotic protein expression in hepatic tissue, as well as pro-inflammatory cytokine production. Moreover, *A. pinnata* ethanolic extract increased anti-inflammatory cytokine production and antioxidant activities in hepatic tissue; thus, it appears to be a promising hepatoprotective agent against toxicants that induce hepatotoxicity.

## Figures and Tables

**Table 1:** Identified compounds from *A. pinnata* ethanolic extract (APE) using GNPS molecular networking database.

Compound	M.F	M.W(Da)
Kaempferol (1)	[C_15_H_10_O_6_] ^+^	286.987
Vitexin (2)	[C_21_H_20_O_10_] ^+^	432.997
Rutin (3)	[C_27_H_30_O_16_] ^+^	611.013
Thiamine (4)	[C_12_H_17_N_4_OS] ^+^	265.047
Tamarixetin (5)	[C_16_H_12_O_7_] ^+^	316.99
Hyperoside (6)	[C_21_H_20_O_12_] ^+^	464.981
Astragalin (7)	[C_21_H_20_O_11_] ^+^	448.99
Quercetin (8)	[C_15_H_10_O_7_] ^+^	302.979

M.F; molecular formula, ^+^ Correlated to [M+H], M.W. (Da); molecular weight (Dalton).

**Table 2:** Initial and final body weights of rats of all experimental groups.

Parameters	Control	Lead	APE	Lead and APE	APE Then Lead	Lead Then APE
Initial BW (g)	121.20 ± 1.28	120.20 ± 1.14	120.30 ± 1.03	120.00 ± 0.85	120.30 ± 1.03	120.40 ± 0.79
Final BW (g)	166.90 ± 2.91 ^a^	101.29 ± 3.39 ^b^	161.30 ± 2.54 ^a^	150.33 ± 0.88 ^a^	154.56 ± 4.19 ^a^	151.50 ± 3.33 ^a^

The values are expressed as the means *±* SE. Values carrying different letters in the same row are significantly different. *Azolla* pinnata ethanolic extract, APE; body weight, BW.

**Table 3:** The effect of lead acetate and/or *Azolla pinnata* ethanolic extract (APE) on liver and kidney function biomarkers in rats.

Parameters	Control	Lead	APE	Lead and APE	APE Then Lead	Lead Then APE
ALT (U/L)	18.00 ± 3.42 ^b^	52.00 ± 0.85 ^a^	19.00 ± 2.09 ^b^	22.75 ± 3.21 ^b^	45. 80 ± 0.88 ^a^	23.75 ± 5.61 ^b^
AST (U/L)	110.40 ± 6.35 ^c^	157.20 ± 8.5 ^a^	110.00 ± 7.78 ^c^	135.00 ± 4.44 ^b^	132.40 ± 3.02 ^b^	137.00 ± 4.69 ^b^
Creatinine (mg/dL)	0.72 ± 0.02 ^b^	1.03 ± 0.05 ^a^	0.62 ± 0.02 ^b^	0.85 ± 0.03 ^b^	0.73 ± 0.04 ^b^	0.74 ± 0.02 ^b^
Urea (mg /dL)	19.41 ± 1.08 ^d^	38.03 ± 0.65 ^a^	21.60 ± 0.66 ^cd^	23.85 ± 1.29 ^c^	27.73 ± 0.42 ^b^	29.72 ± 1.78 ^b^

The values are expressed as the means ± SE. Values carrying different letters in the same row are significantly different. *Azolla pinnata* ethanolic extract, APE; alanine aminotransferase, ALT; aspartate aminotransferase, AST.

**Table 4:** The effects of lead and/or *Azolla pinnata* ethanolic extract (APE) on serum levels of tumor necrosis factor alpha (TNF-α), interleukin 1 beta (IL-1 β) and IL-10 in rats.

Parameters	Control	Lead	APE	Lead and APE	APE Then Lead	Lead Then APE
TNF-α (pg/mL)	85.80 ± 2.10 ^c^	117.75 ± 2.78 ^a^	86.60 ± 2.56 ^c^	97.50 ± 2.96 ^b^	95.0 ± 2.59 ^b^	104.20 ± 2.54 ^b^
IL-1β (pg/mL)	177.20 ± 3.34 ^b^	284.60 ± 3.30 ^a^	169.20 ± 1.93 ^b^	194.75 ± 3.07 ^b^	193.60 ± 5.27 ^b^	199.40 ± 4.23 ^b^
IL-10 (pg/mL)	5.82 ± 0.32 ^b^	1.73 ± 0.23 ^d^	8.92 ± 0.46 ^a^	5.70 ± 0.38 ^b^	3.73 ± 0.71 ^c^	3.725 ± 0.55 ^c^

The values are expressed as the means ± SE. Values carrying different letters in the same row are significantly different. *Azolla pinnata* ethanolic extract, APE; tumor necrosis factor alpha, TNF-α; interleukin 1 beta, IL-1β; interleukin 10, IL-10.

**Table 5:** Effect of lead and/or *Azolla Pinnata* ethanolic extract (APE) on lipid peroxidation and antioxidant biomarkers in hepatic tissues in rats.

Parameters	Control	Lead	APE	Lead and APE	APE Then Lead	Lead Then APE
MDA (nmol/g tissue)	8.85 ± 0.38 ^c^	17.64 ± 0.27 ^a^	10.32 ± 0.44 ^bc^	12.42 ± 0.73 ^b^	12.34 ± 0.16 ^b^	10.95 ± 0.63 ^b^
GSH (mmol/g tissue)	2.84 ± 0.14 ^a^	0.49 ± 0.0 ^c^	2.69 ± 0.11 ^a^	1.22 ± 0.09 ^b^	2.63 ± 0.21 ^a^	1.31 ± 0.30 ^b^
CAT (U/ g tissue)	0.66 ± 0.01 ^a^	0.22 ± 0.0 ^c^	0.63 ± 0.01 ^a^	0.35 ± 0.02 ^b^	0.35 ± 0.02 ^b^	0.33 ± 0.05 ^b^
SOD (U/g tissue)	3.43 ± 0.08 ^a^	1.67 ± 0.06 ^c^	3.57 ± 0.06 ^a^	2.84 ± 0.09 ^b^	3.16 ± 0.18 ^a^	2. 82 ± 0.16 ^b^

The values are expressed as the means ± SE. Values carrying different letters in the same row are significantly different. *Azolla pinnata* ethanolic extract, APE; malondialdehyde, MDA; reduced glutathione, GSH; catalase, CAT; superoxide dismutase, SOD.

**Table 6:** Histopathologic changes induced by lead and ameliorative effect of *Azolla pinnata* ethanolic extract (APE) in hepatic tissues.

Lesions	Control	Lead	APE	Lead and APE	APE Then Lead	Lead Then APE
Vascular and inflammatory changes	-	+++	-	+	+	++
Degenerative and necrotic changes	-	+++	-	+	+	++
Proliferative changes	-	++	-		-	-

- means no changes, + means mild changes, ++ means moderate changes, +++ means severe changes. *Azolla pinnata* ethanolic extract, APE.

**Table 7:** Effects of lead and/or *Azolla Pinnata* ethanolic extract (APE) on the expression of caspase 3 in hepatic tissues of rats.

	Control	Lead	APE	Lead and APE	APE then Lea	Lead then APE
Caspase 3 expression	10.25 ± 0.67 ^e^	70.5 ± 3.73 ^a^	9.20 ± 0.61 ^e^	27.03 ± 1.97 ^c^	18.37 ± 1.01 ^d^	38.83 ± 1.22 ^b^

Values are expressed as the means ± SE. Values carrying different letters in the same row are significantly different. *Azolla pinnata* ethanolic extract, APE.
